# A Photoactivated Gas Detector for Toluene Sensing at Room Temperature Based on New Coral-Like ZnO Nanostructure Arrays

**DOI:** 10.3390/s16111820

**Published:** 2016-10-31

**Authors:** Li-Ko Yeh, Jie-Chun Luo, Min-Chun Chen, Chih-Hung Wu, Jian-Zhang Chen, I-Chun Cheng, Cheng-Che Hsu, Wei-Cheng Tian

**Affiliations:** 1 Graduate Institute of Electronics Engineering, National Taiwan University, Taipei City 10617, Taiwan; likoyeh@gmail.com (L.-K.Y.); life2145@hotmail.com (J.-C.L.); 2 Department of Chemical Engineering, National Taiwan University, Taipei City 10617, Taiwan; b99504035@ntu.edu.tw (M.-C.C.); chsu@ntu.edu.tw (C.-C.H.); 3 Graduate Institute of Applied Mechanics, National Taiwan University, Taipei City 10617, Taiwan; ki781219@gmail.com (C.-H.W.); jchen@ntu.edu.tw (J.-Z.C.); 4 Department of Electrical Engineering, National Taiwan University, Taipei City 10617, Taiwan; iccheng@ntu.edu.tw; 5 Graduate Institute of Biomedical Electronics and Bioinformatics, National Taiwan University, Taipei City 10617, Taiwan

**Keywords:** ZnO gas detector, ZnO coral-like nanostructures, photoactivation, room temperature sensing

## Abstract

A photoactivated gas detector operated at room temperature was microfabricated using a simple hydrothermal method. We report that the photoactivated gas detector can detect toluene using a UV illumination of 2 μW/cm^2^. By ultraviolet (UV) illumination, gas detectors sense toluene at room temperature without heating. A significant enhancement of detector sensitivity is achieved because of the high surface-area-to-volume ratio of the morphology of the coral-like ZnO nanorods arrays (NRAs) and the increased number of photo-induced oxygen ions under UV illumination. The corresponding sensitivity (ΔR/R_0_) of the detector based on coral-like ZnO NRAs is enhanced by approximately 1022% compared to that of thin-film detectors. The proposed detector greatly extends the dynamic range of detection of metal-oxide-based detectors for gas sensing applications. We report the first-ever detection of toluene with a novel coral-like NRAs gas detector at room temperature. A sensing mechanism model is also proposed to explain the sensing responses of gas detectors based on coral-like ZnO NRAs.

## 1. Introduction

In the past decade, metal-oxide gas detectors have attracted substantial interest thanks to their low cost, flexible production, simple application, wide variety of detectable gases, and potential for integration with semiconductors or micro-electro-mechanical systems (MEMS) [1,2]. Various metal-oxide-based materials are used in gas detection because of their numerous advantages, such as high sensitivity and stability and fast response [1,2,3,4].

With the increasing performance of nanotechnology-based chemosensors, various nano-materials, including nanotubes, nanowires, and nanorods, have been investigated for the detection of volatile organic compounds (VOCs). The nanoscale size of these nanomaterials provides excellent chemical, optical, and electrical properties, such as a large surface-area-to-volume ratio. This results in more active sites, an increase in sensitivity, and a lowering of response/recovery times [5]. Among the various methods of nanomaterial synthesis, the hydrothermal synthesis process is highlighted because of its advantages in controllable parameters, low processing temperature, and low cost, and also because it is a possible substitute for flat-plate coating.

High operating temperatures are necessary for conventional metal-oxide-based gas detectors in certain cases [6,7,8,9,10]. However, the need to operate these detectors at high temperatures requires high power, which limits their potential usage. Recently, several approaches have been proposed to reduce the operating temperature of detectors, including using doped metals in metal-oxide materials e.g., Pd-catalyzed TiO_2_, TiO_2_-doped ZnO, Pd-doped SnO_2_, and Au-modified ZnO [11,12,13,14,15]. In addition, improving the thermal isolation using MEMS technology [16], using alternative nanosensing materials [17], and incorporating ultraviolet (UV) illumination during detection [18,19] also decrease the operating temperature of detectors. Among these techniques, the application of UV illumination on metal-oxide detectors is one of the most promising methods for achieving room temperature gas sensing.

ZnO is an important semiconductor material with a wide and direct band gap (E_g_ = 3.37 eV). It has been widely used with high sensitivity for the detection of various gases [20,21,22,23,24,25,26]. It is also suitable for the synthesis of nanomaterials and has a broad UV absorbing spectrum.

Toluene (C_6_H_5_CH_3_) is a toxic VOC with a high octane number. It is widely used as a solvent and additive in petrol and is also an important raw material in the organic chemical industry. However, gaseous toluene is highly toxic and originates from paint and varnish thinners commonly used in the construction industry [27]. In addition, toluene is a flammable gas [28]. Detecting toluene as accurately as possible, especially at room temperatures and low concentrations, is of great importance. However, most recently published literature on detecting toluene focuses on high-temperature ZnO-based sensitive gas detectors [7,8,10,29]. Having sufficient reaction energy for oxidization in practice, these detectors are typically operated above 200 °C in order to achieve high-sensitivity operation with a fast sensing response.

In this work, a simple and low-cost hydrothermal process is adopted to synthesize coral-like ZnO. The high sensitivity of the ZnO detector to toluene is explained on the basis of nanowire bridges for electron transfer for sensing gas molecules. All operating processes of the ZnO detectors occur at room temperature (<30 °C). It is our goal to develop an universal detector for gas chromatography system [30]. In this study, toluene was chosen as a representative VOC to demonstrate the feasibility of the proposed nanostructured detectors.

## 2. Materials and Methods

### Characterization

In this study, the morphology of ZnO nanostructures were observed by scanning electron microscope (JEOL Ltd., Akishima, Tokyo, Japan) while X-ray diffraction (PANalytical Ltd., Almelo, The Netherlands) was used to check the crystalline structure of the prepared samples. An UV-lamp (TROTEC Ltd., Heinsberg, Germany) had been used as a 365 nm light source for UV illumination experiments. An UV absorbing filter (HOYA, Shinjuku, Tokyo, Japan) and a power meter (OPHIR Ltd., North Andover, MA, USA) were utilized to decrease the UV intensity to operate the UV illumination at 2 μW/cm^2^. The ZnO thin film was deposited by the sputter system (Kao Duen Ltd., Taipei, Taiwan). To characterize the device performances, the optical reflectance spectra were measured using a standard spectrometer (JASCO, Hachioji, Tokyo, Japan) and the temperature of devices was monitored by an infrared (IR) detector (FLIR, Wilsonville, OR, USA). 

**Figure 1:**
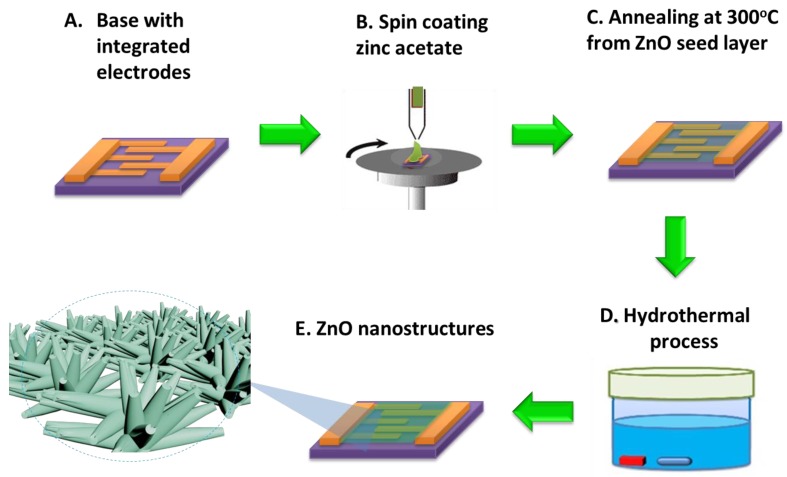
Synthesis of ZnO nanostructures on integrated electrodes.

A two-layer of metal with 20 nm Ti and 200 nm Au was deposited sequentially on the oxidized Si wafer followed by lithography and Au etchant to form the interdigitated electrodes. These Si chips with interdigitated electrodes were served as the device platform and either the ZnO thin film or ZnO NRAs was fabricated on top of the electrodes as the sensing material. 100 nm ZnO thin films were deposited by using a sputter to serve as a reference for comparison. The growth conditions were optimized and the gas mixture in a sputter is argon, oxygen and nitrogen fixed at 20, 5 and 45 mTorr, respectively. The rf power to sputter the ZnO target was 125 W at room temperature. The flower-like and coral-like ZnO NRAs were synthesized using a hydrothermal process as shown in Figure 1. The ZnO seed-layer was prepared by 25 mM zinc acetate (Zn(CH_3_COO)_2_, 99% purity, Sigma, St. Louis, MO, USA) coating as shown in Figure 1b,c. The addition of vitamin C to the solution causes the inhibition in the growth rate of ZnO crystal along the [0001] direction [31]. The water used in all experiments was deionized (DI) and had a resistivity of 17 MΩcm. The flower-like ZnO NRAs were grown in an aqueous solution containing 10 mM zinc nitrate hexahydrate (Zn(NO_3_)_2_·6H_2_O, 98% purity, Sigma, St. Louis, MO, USA) and 20 mM hexamethylenetetramine (HMTA, C_6_H_12_N_4_, ≥99% purity, Sigma, St. Louis, MO, USA) at 95 °C for 10 min with a rotor speed of 500 rpm. The coral-like ZnO NRAs were grown in an aqueous solution containing 10 mM zinc nitrate hexahydrate mixed with 20 mM HMTA and 0.5 mg/L vitamin C at 95 °C for 10 min with a rotor speed of 300 rpm and the ZnO layers were placed facing downward, positioned at the bottom of the beaker [32]. The ZnO NRAs were synthesized using a one-step and low-cost hydrothermal process. Finally, NRAs with different morphologies were cleaned with ethanol and DI water in ambient air. A compact ZnO sensing film was coated between the gaps of the interdigitated electrodes.

**Figure 2:**
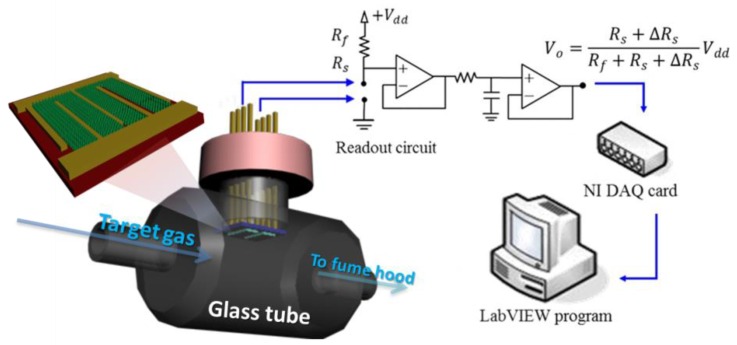
Schematic diagram of gas sensing measurement systems.

Figure 2 shows the setup of the measurement system and the structure of the device. A simple custom-made testing platform, comprising the ZnO gas detector, a gas generation system [4,33], a power supply (Keithley, Cleveland, OH, USA), a high-precision ohmmeter (Keithley), and an IR detector were constructed to evaluate the functionality of the proposed detectors. A gas detector was mounted on a printed circuit board, which was used as the chamber for the experiments. The room temperature (28 °C) and humidity (60% RH) of the test chamber were well controlled during detector testing. The readout circuit was a voltage divider consisting of a DC power supply, a reference resistor, a detector resistor, and a single operational amplifier (LF 353). When the system injected gas into the testing chamber, the resistance of the detector changed as a result. The output voltage was measured at the non-inverting input of the LF 353 employed. Ambient air was pumped through an air compressor at a flow rate of 10 mL·min^−1^ and served as the carrier gas. The change of sensing resistance of our detector upon exposure of the target gas was recorded. The changes in detector resistance were measured in real time with the readout circuit and delivered to a signal acquisition system.

## 3. Results

**Figure 3:**
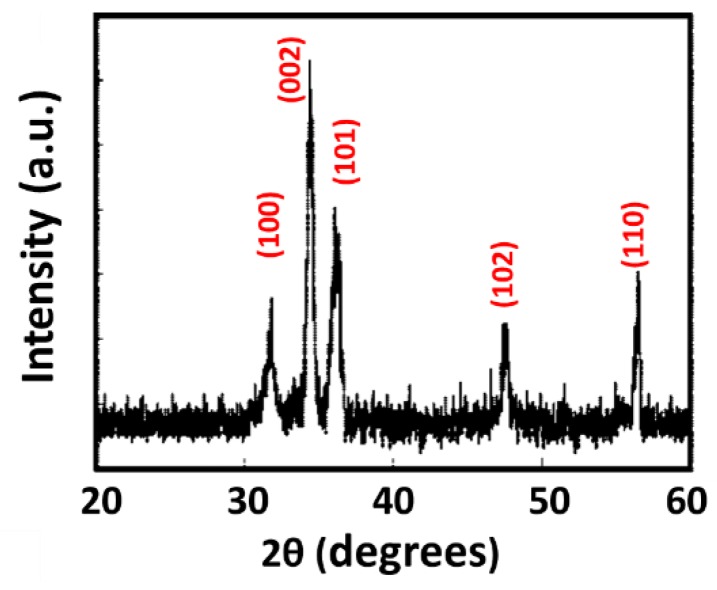
XRD patterns of ZnO detectors.

Figure 3 shows X-ray diffraction (XRD) patterns of the ZnO nanostructures without any annealing. A clear peak (degree) at 2*θ* = 34.4° with a strong reflection from the (002) plane appears, demonstrating the occurrence of polycrystallization. Several additional peaks, including reflections from the (100), (101), (102), and (110) orientation planes, were indexed to hexagonal wurtzite ZnO with lattice constants of a = 0.3250 nm and c = 0.5206 nm. No other impurities were observed, indicating a high degree of crystallization of ZnO. The sizes of the crystallite of the prepared ZnO nanostructures were calculated using the full width at half maximum of the (002) peak employing Scherrer’s formula [34].

**Figure 4:**
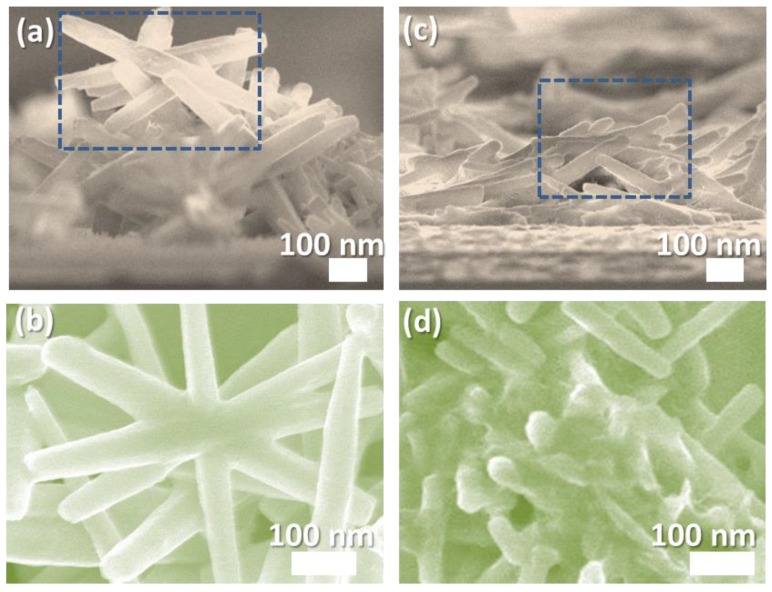
(**a**) Cross-sectional and (**b**) top-view SEM images of a flower-like ZnO NRAs; (**c**) Cross-sectional and (**d**) top-view SEM images of a coral-like ZnO NRAs.

Figure 4 shows the images of the flower- and coral-like ZnO NRAs, recorded using field-emission scanning electron microscopy. Figure 4a,c are cross-sectional SEM images. Figure 4a shows that flower-like ZnO nanostructures have a greater brightness in contrast to the coral-like structures shown in Figure 4c. The morphology of the ZnO nanostructures shows that the thicknesses of the flower- and coral-structures are approximately 1 μm and 500 nm, respectively. 

The measurements of thicknesses were collected from two independent batches using an image analysis software (ImageJ, Bethesda, MD, USA) in SEM analysis. The diameter of both nanowires is approximately 60 nm. Figure 4c shows that the ZnO nanowires form a bridge conformation on the ZnO films. Figure 4b,d show top-view SEM images which were enlarged from the rectangular areas in Figure 4a,c.

**Figure 5:**
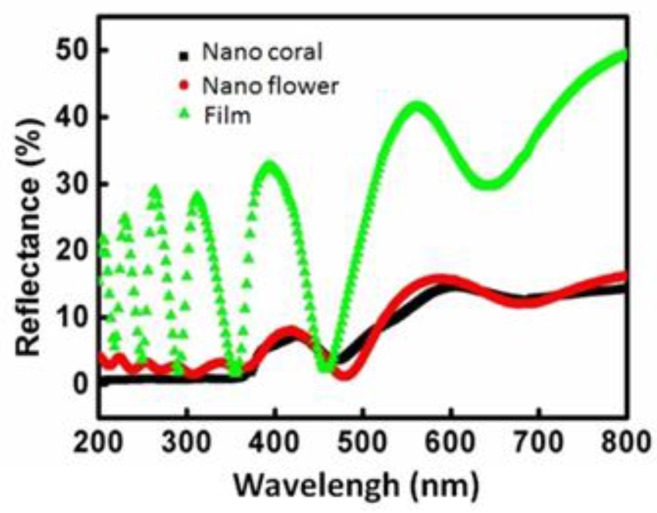
Specular reflectance measured on the device with film for flower-like and coral-like nanostructure surfaces.

Figure 5 shows spectra of specular reflectance measured on the three detectors with different surface conditions: with a ZnO thin-film layer (100 nm), with flower-like ZnO, and with coral-like ZnO. The spectra were obtained at an incidence angle of 5° and the wavelengths ranged from 300 nm to 800 nm. The ZnO layer has an effective refractive index of approximately 2.02 and an extinction coefficient of nearly zero [35]. The values of reflectance fluctuate because the incident waves reflect off the top surface of the interdigitated electrode. The thickness of the film is of the order of the wavelength of the incident wave, thus causing typical thin-film interference. For the devices with a ZnO nanostructure, the reflection is almost constant from 400 nm to 700 nm, but gradually decreases as the wavelength approaches the UV range close to 400 nm. From Figure 5, the addition of a nanostructure layer leads to an even lower reflectance. Unlike the reflectance oscillation observed in the device with a thin-film layer, the reflectance of the devices with the nanostructured layers stays below 5% for all the studied wavelengths, therefore exhibiting superior anti-reflectance (AR) performance. This AR characteristic increases the UV absorption efficiency of devices with nanostructured layers.

**Figure 6:**
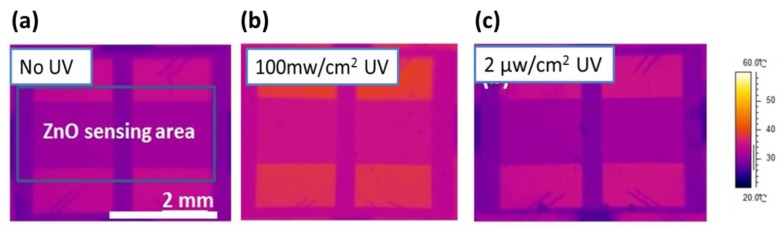
IR images of nanoscale coral-like detectors: (**a**) no UV illumination; (**b**) the device illuminated with 100 mW/cm^2^ UV illumination; and (**c**) with 2 μW/cm^2^ UV illumination.

In order to measure the operating temperature of the UV photoactivated detector, an IR detector, was constructed to evaluate the functionality of the proposed detectors. An IR detector was used to monitor the temperature distribution of the detector, to confirm that experimental UV illumination (2 μW/cm^2^) had negligible effect of temperature during testing, as shown in Figure 6. Figure 6a shows an IR image of the coral-like detector with no illumination and Figure 6b shows a control group with 100 mW/cm^2^ high intensity UV illumination. It can be seen that the temperature of the devices increases as a result of high-intensity (100 mW/cm^2^) UV illumination, as shown in Figure 6b. However, our device only requires low-intensity UV illumination (2 μW/cm^2^) to stimulate sufficient photo-induced oxygen ions at room temperature.

The absorption spectrum of our photoactivated sensing material falls in the region where UV-emission electrons can be generated by the UV illumination, which is helped by the adsorption of oxygen. This increases the number of photo-induced oxygen ions on the surface of the sensing material. These increased oxygen ions typically appear at elevated temperatures in other studies and can enhance the proposed performance of the detector at room temperature.

The ratio for a distinguishable change in resistance is defined as the sensitivity, ΔR/R_0_, where R_0_ is the initial resistance. The sensing response of the ZnO detectors to toluene was higher at higher gas concentrations because there were more toluene molecules that could respond to the oxygen ions over the ZnO surface, compared to reactions with fewer toluene molecules.

**Figure 7:**
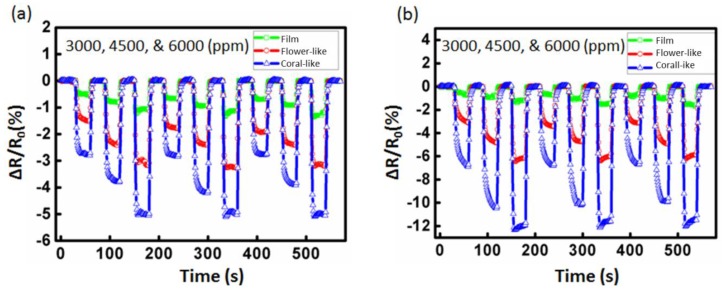
Sensitivity (ΔR/R_0_) curves of the detector for different concentrations of toluene gas: (**a**) non-photoactivated and (**b**) photoactivated with 2 μW/cm^2^ UV illumination.

Detailed characterization results for the ZnO film and flower-like and coral-like nanostructure detectors are shown in Figure 7. For 3000, 4500, and 6000 ppm of toluene injected onto the gas detectors separately, the transient sensitivity values of the ZnO-based gas detectors to toluene with no UV illumination at room temperature are shown in Figure 7a. Before UV illumination, the film detector’s average values of sensitivity to toluene gas were 0.51% (3000 ppm), 0.76% (4500 ppm), and 1.04% (6000 ppm). The flower-like detector’s average values of sensitivity to toluene were 1.48% (3000 ppm), 2.31% (4500 ppm), and 3.13% (6000 ppm). The coral-like detector’s average sensitivity to toluene was 2.73% (3000 ppm), 4.01% (4500 ppm), and 5.27% (6000 ppm), respectively, as shown in Figure 7a. The coral-like detectors demonstrated the greatest enhancement of sensitivity, with a >500% increase (from 0.51% to 2.73% at 3000 ppm; from 0.76% to 4.01% at 4500 ppm; from 1.04% to 5.27% at 6000 ppm) compared to film detectors at room temperature.

After applying 2 μW/cm^2^ UV illumination to the ZnO surface, the increase in photo-induced electrons from ZnO promoted the formation of oxygen ions. These additional oxygen ions were highly reactive and resulted in the enhanced sensing response. Therefore, the sensitivity of the three detectors was increased. The film detector’s average values of sensitivity to toluene gas increased to 0.65% (3000 ppm), 0.98% (4500 ppm), and 1.32% (6000 ppm). The flower-like detector’s average values of sensitivity to toluene increased to 3.03% (3000 ppm), 4.67% (4500 ppm), and 6.20% (6000 ppm). The coral-like detector’s average values of sensitivity to toluene increased to 6.53% (3000 ppm), 10.02% (4500 ppm), and 12.57% (6000 ppm), following UV illumination of the ZnO surface. The detector’s sensitivity and the corresponding enhancement ratio for toluene under UV illumination are shown in Figure 7b. The photoactivated coral-like detectors saw enhancements of sensitivity of >950% (from 0.65% to 6.53% at 3000 ppm; from 0.98% to 10.01% at 4500 ppm; from 1.32% to 12.57% at 6000 ppm) compared to film detectors with 2 μW/cm^2^ UV illumination.

**Figure 8:**
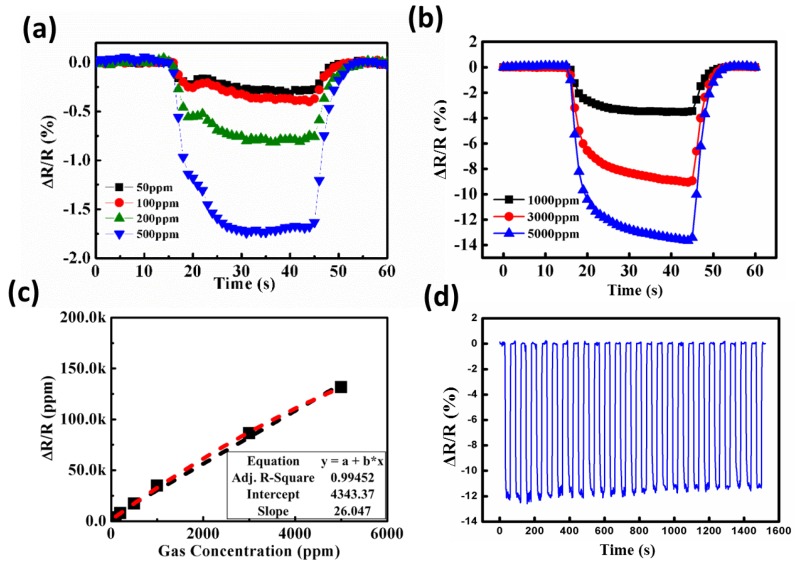
Sensitivity (ΔR/R_0_) of coral-like nanostructure gas detectors for toluene concentrations in ranges (**a**) 50–500 ppm and (**b**) 1000–5000 ppm; (**c**) Fitted curve of the coral-like ZnO detector to toluene with different concentrations; (**d**) Stability performances of coral-like detectors with 6000 ppm toluene were conducted over 25 cycles under UV illumination of 2 μW/cm^2^.

In order to achieve optimal operating conditions, the responses of coral-like ZnO gas detectors to toluene were tested for concentrations ranging from 50 ppm to 5000 ppm. Figure 8 shows the transient sensitivity characteristics of the coral-like ZnO detectors to toluene for various toluene concentrations with 2 μW/cm^2^ UV illumination at room temperature. With increasing concentration, the gas detector exhibited outstanding sensitivity to toluene. A relatively good linear response–concentration relationship for the concentration range 50–500 ppm appears in Figure 8a and in the range 1000–5000 ppm in Figure 8b. The fitted curve of the sensitivity to toluene with different concentrations is shown in Figure 8c for the range 100–5000 ppm. The correlation coefficient R of the fitted curve is 0.99452, implying a good match between the fitted curve and the experimental data, and a linear detector response. To perform the stability tests, toluene with a concentration of 6000 ppm was applied onto the gas detector, and the stability performances of coral-like detectors under an UV illumination of 2 μW/cm^2^ were conducted. As shown in Figure 8d, the value of sensitivity slightly decreases from 12.23 to 11.66 initially (4.67% from 1st to 5th cycle). The performance reaches almost steady state after 10 cycles and the value of sensitivity changes only 1.71% from 11th to 25th cycle. It is demonstrated that our ZnO detector is relatively stable over multiple tests.

## 4. Discussion

**Figure 9:**
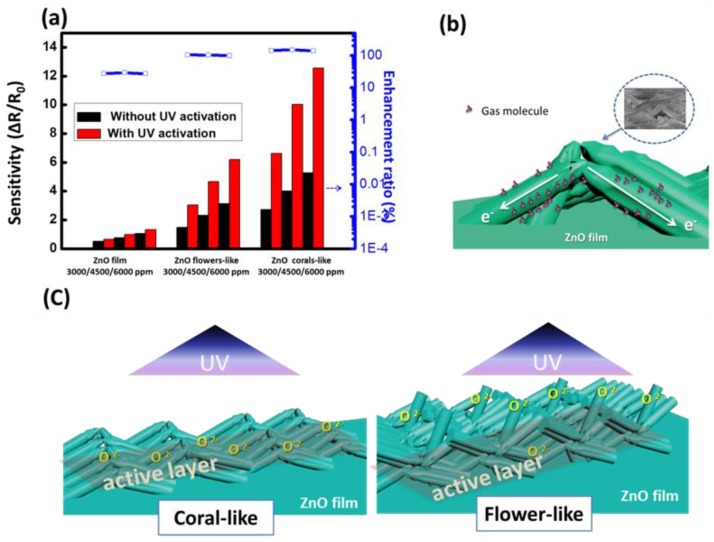
(**a**) Sensitivity and enhancement ratio of the detector to toluene; (**b**) Schematic illustration of ZnO-nanowire bridges over the ZnO film. The inset in the upper-right corner is the real SEM image; (**c**) Simple model for ZnO flower-like and coral-like detectors with 2 μW/cm^2^ UV illumination; the rectangle shows the effective active layer for gas sensing.

The value of the detector sensitivity and the corresponding enhancement ratio for toluene are shown in Figure 9a. The average enhancement ratios of sensitivity with UV activation for the three surface structures for toluene injection were 27.78% (film), 101.66% (flower-like), and 143.76% (coral-like), respectively. The devices based on coral-like ZnO with the nanowire morphology landslide to form bridges over the ZnO film, as shown in Figure 9b. The surface conditions of the nanostructures are crucial to their gas sensing properties. It is worth noting that most of the nanowires on the coral-like surface were lying above the patterned electrodes as shown in Figure 4c. We propose a mechanism for the operation of nanowires on the surface of coral-like detectors. In summary, the gas sensing process is strongly related to the surface reactions of ZnO. Devices based on bridging nanowires have been realized and demonstrated with enhanced performances due to the surface morphology [36,37]. ZnO nanowires grown on patterned electrodes have many nanowire–nanowire junctions on coral-like detectors. Nanowire-nanowire junctions of coral-like surface constitute an effective mechanism of electron transfer through bridged nanowire devices across electrode surfaces. The access nanowires on the trenches of the coral-like top surface can be regarded as a fast electron-transfer channel with a gas-molecule agglomeration on the ZnO surface.

When an UV illumination was applied to the nanostructured ZnO, electron–hole pairs will be generated. The holes migrate to the surface and recombine with the O_2_ species adsorbed on the surface; the unpaired electrons are collected at the anode under an applied voltage, which leads to the increase of the nanowires conductivity [26]. With an UV illumination of 2 μw/cm^2^, more electrons were generated on the surface of ZnO. It is therefore that the initial resistances (R_0_) of the ZnO thin film device, flower-like and coral-like devices were reduced from 2 M to 800 k, 1.5 M to 800 k and 1.6 M to 700 k, respectively. The increased electrons also enhanced the oxidization of the surface organic contaminant. Hence, the surface of the sensing layer was cleaned to enhance gas adsorption. These results indicate that UV illumination can improve sensing response and broaden the detectable range for more gas species in gas detector applications [4]. However, only increased oxygen ions on the active layer of detectors contribute to the enhancement of sensitivity. Both flower-like and coral-like nanostructures has similar reflection spectrum in the UV range as shown in Figure 5, it is assumed that the amount of effective oxygen ions generated by UV illumination for flower-like and coral-like detectors are similar.

Following UV irradiation, the sensitivity of the photoactivated detector increased on the ZnO surface, particularly on coral-like surfaces. We propose a simple model to explain this. Figure 9c shows the schematic diagram of the oxygen ions generated with UV illumination on the flower- and coral-like based detectors. For flower-like based detectors, it is assumed that oxygen ions are generated mostly on the upper part of the flower-like structure. However, the flower-like detector has few oxygen ions close to the substrate. Most of the generated oxygen ions do not contribute to the reaction on the ZnO surface. The rectangle shows the effective active layer for gas sensing. Only the increased oxygen ions in the active layer contribute to the resistance change for gas sensing. More oxygen ions in the active layer result in a greater change in the resistance. The coral-like detector shows the greatest increase in oxygen ions on an active layer close to the ZnO film substrate. This results in coral-like detectors having higher sensitivity than flower-like detectors. 

## 5. Conclusions

In summary, our study demonstrated the operation of a new type of photoactivated, room-temperature coral-like, ZnO-based gas detector, fabricated using a simple hydrothermal approach. X-ray diffraction results indicated that pure ZnO with a hexagonal wurtzite structure was generated. The as-prepared ZnO with a coral-like morphology exhibited good sensitivity toward toluene when operating at room temperature. The effects of UV activation on ZnO-based detectors were also studied. With illumination by UV light, significant UV-induced oxygen ions were generated on the ZnO surface, which enhanced the sensitivity of the detectors. Taking advantage of the geometry of ZnO coral-like structures, the great enhancement of sensitivity of the coral-like detectors was confirmed. In this study, we proposed that coral-like ZnO has a high sensitivity as a result of an extraordinary electron transfer mechanism. The corresponding sensitivity (ΔR/R_0_) of the coral-like ZnO detector was enhanced by approximately 1022% compared to thin-film detectors at toluene concentrations of 4500 ppm with 2 μW/cm^2^ UV illumination.
